# Global Proteomic Analysis of *Listeria monocytogenes’* Response to Linalool

**DOI:** 10.3390/foods10102449

**Published:** 2021-10-14

**Authors:** Zhipeng Gao, Weiming Zhong, Ting Liu, Tianyu Zhao, Jiajing Guo

**Affiliations:** 1 Hunan Engineering Technology Research Center of Featured Aquatic Resources Utilization, College of Animal Science and Technology, Hunan Agricultural University, Changsha 410128, China; zhongweiming2021@163.com (W.Z.); z1583025825@163.com (T.Z.); 2 Hunan Agriculture Product Processing Institute, Hunan Academy of Agricultural Sciences, Changsha 410125, China; ltchangsha98@163.com; 3 Key Laboratory of Agro-Products Processing, Ministry of Agriculture and Rural Affairs of P. R. China, Institute of Food Science and Technology CAAS, Beijing 100193, China

**Keywords:** linalool, *Listeria monocytogenes*, antimicrobial, proteomics

## Abstract

*Listeria monocytogenes* (LM) is one of the most serious foodborne pathogens. Listeriosis, the disease caused by LM infection, has drawn attention worldwide because of its high hospitalization and mortality rates. Linalool is a vital constituent found in many essential oils; our previous studies have proved that linalool exhibits strong anti-*Listeria* activity. In this study, iTRAQ-based quantitative proteomics analysis was performed to explore the response of LM exposed to linalool, and to unravel the mode of action and drug targets of linalool against LM. A total of 445 differentially expressed proteins (DEPs) were screened out, including 211 up-regulated and 234 down-regulated proteins which participated in different biological functions and pathways. Thirty-one significantly enriched gene ontology (GO) functional categories were obtained, including 12 categories in “Biological Process”, 10 categories in “Cell Component”, and 9 categories in “Molecular Function”. Sixty significantly enriched biological pathways were classified, including 6 pathways in “Cell Process”, 6 pathways in “Environmental Information Processing”, 3 pathways in “Human Disease”, 40 pathways in “Metabolism”, and 2 pathways in “Organic System”. GO and Kyoto Encyclopedia of Genes (KEGG) enrichment analysis together with flow cytometry data implied that cell membranes, cell walls, nucleoids, and ribosomes might be the targets of linalool against LM. Our study provides good evidence for the proteomic analysis of bacteria, especially LM, exposed to antibacterial agents. Further, those drug targets discovered by proteomic analysis can provide theoretical support for the development of new drugs against LM.

## 1. Introduction

The prevention and control of foodborne pathogens is always an urgent need for food safety and human health worldwide [1]. *Listeria monocytogenes* is listed as one of the most serious foodborne pathogens by the World Health Organization (WHO) [2]. It is widely distributed in nature and can survive in many extreme environments such as high salt, low temperature, low pH, and so on [3,4]. Listeriosis caused by LM infection is a serious food-borne zoonotic disease with high hospitalization and mortality rates [5,6,7,8]. The clinical manifestations mainly include meningitis, septicemia, and endocarditis. Pregnant women, newborns, the elderly, and people with weakened immunity are susceptible groups. Among them, pregnant women are more likely to suffer from listeriosis, and severe cases can even cause premature birth, stillbirth, and neurological diseases in the offspring [3,9]. Thus, the control and prevention of LM has become a crucial issue all over the world.

Chemical antimicrobial agents are usually used for the prevention of LM in the food industry. However, today more and more consumers are likely to pursue “green and natural” foods with few or no chemicals. Yet natural antimicrobial agents, especially essential oils or their ingredients, have become a popular research area [10,11,12,13]. Linalool (3,7-dimethyl-1,6-octadien-3-ol) is a vital constituent found in many essential oils with good antibacterial activity against different kinds of microorganisms. It is generally recognized as a safe (GRAS) food additive [14]. In our previous study, we demonstrated that linalool was a major component in citrus essential oils and exhibited strong antibacterial activity against LM—the ZOI, MIC, and MBC values were 39.58 ± 0.74 mm, 0.5% (*v*/*v*), and 1% (*v*/*v*), respectively—and it exhibited significant anti-biofilm activity by the dispersal and killing of cells in biofilm [15,16,17], but little is known about its mode of action against LM.

Recently, with the rapid development of omics-technology, many omics, such as genomics, transcriptomics, and proteomics, have been used as effective research tools for microbiology study [18,19,20]. Among them, proteomics is often used to investigate bacterial behavior under different environmental conditions in protein levels [13,21]. Research focused on the theme of “changes of microbial proteomics after drug treatment” has become a hot topic. Especially in “antibiotic treatment” fields, some research groups have used proteomics technology on the following research objects: erythromycin against *Streptococcus suis* [22], vancomycin against *Streptomyces coelicolor* [23], daptomycin against *Staphylococcus aureus* [24], bostrycin against *Mycobacterium tuberculosis* [25], emodin against *Haemophilus parasuis* [26], oxytetracycline against *Edwardsiella tarda* [27], etc.

Thus, in this study, we performed a global protein analysis by using iTRAQ (isobaric tags for relative and absolute quantitation)-based quantitative proteomic technology [28,29] to explore how LM responds to the treatment of linalool, and to unravel the mode of action and the drug targets of linalool against LM. We hope this continuous research will provide more of a theoretical basis for the prevention and control of LM.

## 2. Materials and Methods

### 2.1. Bacterial Strains

The LM (ATCC 19115) strain was obtained from Guangdong Microbiology Culture Center (GMCC, Guangdong, China) and stored at −80 °C.

### 2.2. Linalool

Linalool solution (95%) was purchased from Sigma-Aldrich (Sigma-Aldrich, Burlington, MA, USA). The antimicrobial activity of linalool was tested in our previous study, which showed the MIC value was 0.5% (*v*/*v*) [16].

### 2.3. Treatment of LM by Linalool

LM was grown in Brain Heart Infusion broth (BHI, Guangdong Huankai Microbial, Guangdong, China) at 37 °C with shaking overnight and transferred to fresh BHI broth at a ratio of 1:50. When the growth state of LM reached the logarithmic phase, linalool at a concentration of 4 × MIC was added to the bacterial solution and cultured at 37 °C with shaking for 8 h. After that, the cells were centrifuged (4000× *g*, 10 min) and washed three times with sterile PBS. Finally, the cells were collected for both flow cytometry and proteomics assay. For the proteomics assay, cells were frozen in liquid nitrogen for 3 h and then stored at −80 °C before protein extraction.

### 2.4. iTRAQ-Based Quantitative Proteomics Analysis

#### 2.4.1. Protein Extraction, Digestion, and Labeling with iTRAQ Reagents

The treatment of LM cells was mentioned in Section 2.3. According to the manufacturer’s protocol ( from the Majorbio company, Shanghai, China), LM cells were re-suspended with a lysis buffer (cocktail of 1% SDS and 8 M urea) in the ratio of 1:8, sonicated (Fielda-650D, Jiangsu TRON Intelligent Technology Co., Ltd., Jiangsu, China) for 4 min, and incubated on ice for 30 min. The lysates were centrifuged at 12,000× *g* at 4 °C for 15 min and the supernatants were collected. The concentration of protein was determined by bicinchoninic acid (BCA) assay. Protein digestion was carried out according to the standard procedure and the resulting peptide mixture was labeled by using 8-plex iTRAQ reagents according to the instructions (Applied Biosystems, MA, USA). For 8-plex labeling, each iTRAQ reagent was dissolved in 50 μL of isopropanol, added to the peptide mixture, and incubated at room temperature for 2 h. The samples were labeled as (HN12-1)-115, (HN12-2)-116, (HN12-3)-117, (PG45-1)-118, (PG45-2)-119, and (PG45-3)-121. Finally, all the samples were mixed together and vacuum dried before LC-MS/MS Analysis.

#### 2.4.2. Chromatographic Separation and LC-MS/MS Analysis

Samples were re-suspended by loading buffer (ammonium hydroxide solution containing 2% acetonitrile, pH 10) and separated by high-pH reversed phase liquid chromatography (RPLC, Waters, Milford, MA, USA). The gradient elution was performed on a high pH RPLC column at 400 μL/min with the gradient increased for 66 min. Twenty fractions were collected from each sample. LC-MS/MS analysis was performed by a Q Exactive mass spectrometer (Thermo Fisher Scientific, Waltham, MA, USA) coupled with an Easy-nLC 1200 (Thermo Fisher Scientific, Waltham, MA, USA) in the data-dependent mode. Survey full-scan MS spectra were acquired at a mass resolution of 70 K, followed by 20 sequential high energy collisional dissociation LC-MS/MS scans at a resolution of 17.5 K. One micro-scan was recorded by using a dynamic exclusion of 18 s.

#### 2.4.3. Proteomic Analysis

All the LC-MS/MS spectra were searched by using the Protein Discoverer Software (ProteomeDiscoverer^TM^ Software 2.4, Thermo Fisher Scientific, Waltham, MA, USA) against the Mycoplasma database. The highest score for a given peptide mass was used to identify parent proteins. The parameters for protein searching were as follows: tryptic digestion with up to two missed cleavages, carbamidomethylation of cysteine as the fixed modification, and oxidation of methionine and protein N-terminal acetylation as variable modifications. Peptide spectral matches were validated based on q-values at a 1% false discovery rate.

### 2.5. Flow Cytometry Analysis

Flow cytometry analysis was carried out to investigate the effects of linalool on LM. After treatment as mentioned in Section 2.3, the bacterial cells were collected by centrifugation and adjusted to the concentration of 1 × 10^6^ cfu/mL; then, different staining procedures were proceeded as follows [30,31].

#### 2.5.1. Membrane Permeability

Thiazole orange (TO, Sigma-Aldrich, Burlington, MA, USA) and propidium iodide (PI, Sigma-Aldrich, Burlington, MA, USA) were used to evaluate the membrane permeability of the cells. For TO staining, 1 μL of TO solution was added to 1 mL bacterial suspensions (final concentration of TO: 10 μg/mL in DMSO), and then incubated at room temperature for 15 min. For PI staining, 1 μg PI was added to 1 mL bacterial suspensions (final concentration of PI: 1 μg/mL in PBS) and then incubated at 37 °C for 15 min.

#### 2.5.2. Membrane Potential

Bis-1,3-dibutylbarbutiric acid (BOX, Sigma-Aldrich, MA, USA) and PI were used to evaluate the membrane potential of cells. For BOX staining, 2.5 μg of BOX was added to 1 mL bacterial suspensions (final concentration of BOX: 2.5 μg/mL in PBS with 4 mM EDTA), and then incubated at 37 °C for 15 min. For PI staining, the procedure was the same as mentioned in Section 2.5.1.

#### 2.5.3. Efflux Activity 

Ethidium bromide (EB, Sigma-Aldrich, Burlington, MA, USA) was used to evaluate the efflux activity of cells. For EB staining, 10 μg of EB was added to 1 mL bacterial suspensions (final concentration of EB: 10 μg/mL in DMSO), and then incubated at 37 °C for 15 min.

#### 2.5.4. Respiratory Activity

5-cyano-2,3-ditolyl tetrazolium chloride (CTC, Sigma-Aldrich, MA, USA) was used to evaluate the respiratory activity of cells. For CTC staining, 5 mM of CTC was added to 1 mL bacterial suspensions (final concentration of CTC: 5 mM in PBS with 1% (*w*/*v*) glucose), and then incubated at 37 °C with shaking (250 rpm) for 30 min.

After finishing these staining procedures, samples were washed with PBS three times, the concentration of the bacterial suspension was adjusted to about OD600 = 0.1, and the samples were placed on ice for flow cytometry analysis by a flow cytometer (BD Accuri C6 plus, BD, Franklin Lakes, NJ, USA).

### 2.6. Statistical Analysis

All the experiments were performed in triplicate. Statistical analysis was performed by GraphPad Prism 7.0 for *t*-tests. All asterisks indicate significant differences (*p* < 0.05).

## 3. Results

### 3.1. Quality Assessment of Proteomics Sequencing

**Figure 1:**
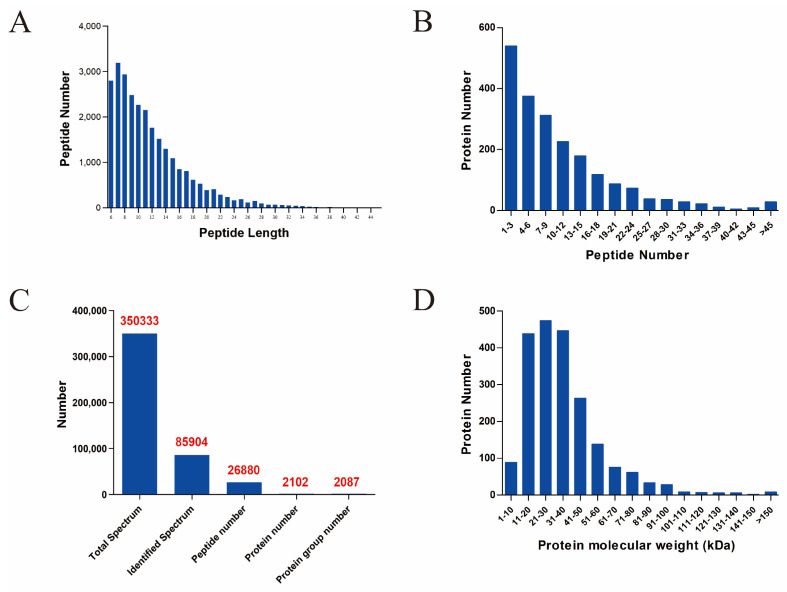
The quality assessments of proteomics sequencing. (**A**) Histogram of peptide length distribution. The abscissa represents the range of peptide length, and the ordinate represents the number of peptides of the corresponding length. (**B**) Histogram of peptide quantity distribution. The abscissa represents the range of the number of peptides covering the proteins, and the ordinate represents the number of the proteins. (**C**) Statistical histogram of different types of identified proteins. (**D**) Histogram of protein molecular weight distribution. The abscissa represents the distribution range of protein molecular weight, and the ordinate represents the number of proteins corresponding to the molecular weight.

As shown in Figure 1, a total of 26,880 peptides and 2102 proteins were identified by proteomics sequencing (Figure 1C). The lengths of the peptides were mainly between 6–20 amino acids, among which 7 was the most common (Figure 1A). The number of peptides that make up proteins was mainly concentrated between 1–21, among which 1–3 was the most common (Figure 1B). Among those identified proteins, the molecular weight was mainly concentrated between 11–60 kDa, among which 21–30 kDa was the most common (Figure 1D).

### 3.2. Functional Annotation and Analysis of Proteins

**Figure 2:**
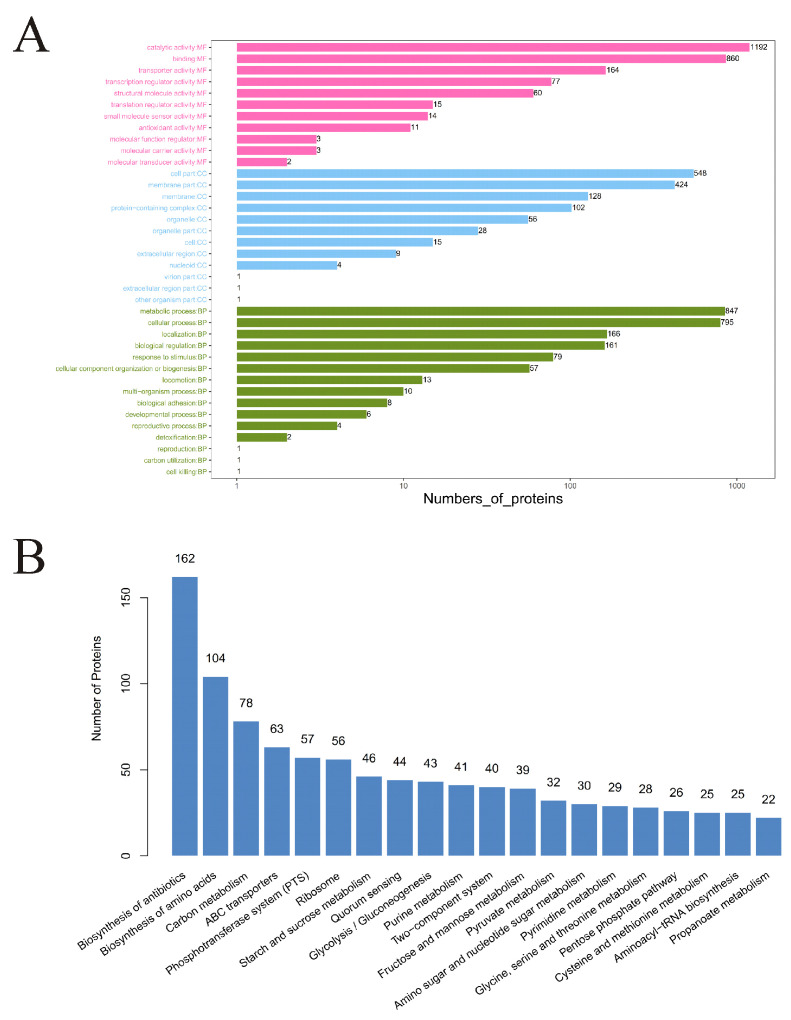
The annotation and analysis of all the identified proteins. (**A**) Annotation based on GO function classification. Each column represents a secondary classification. The ordinate represents the secondary classification term of GO, and the capital letters in front of the term represent the following categories: BP, biological process; CC, cellular component; MF, molecular function. (**B**) The top 20 KEGG pathways with the largest number of proteins. The abscissa represents the name of pathways, and the ordinate represents the number of proteins within each pathway.

After quality assessment, GO and KEGG databases were used to annotate and analyze the functions of the identified proteins to explore their biological pathways and functions. As shown in Figure 2A, Biological Processes, Cell Components, and Molecular Functions (within which all the identified proteins were included) were obtained by GO classification annotation. Among “Molecular Functions”, the top five were the catalytic activity, binding, transporter activity, transcription regulator activity, and structural molecule activity, and the number of proteins involved were 1192, 860, 164, 77, and 60, respectively. Among “Cell Components”, cell part, membrane part, membrane, protein-containing complex, and organelle were the top five, and the number of proteins involved were 548, 424, 128, 102, and 56, respectively. Among “Biological Processes”, metabolic process, cellular process, localization, biological regulation, and response to stimulus were the top five, and the number of proteins involved were 847, 795, 166, 161, and 79, respectively.

As shown in Figure 2B, the top 20 KEGG pathways with the largest number of proteins involved were obtained by KEGG classification annotations. Among them, the top 10 pathways were biosynthesis of antibiotics, biosynthesis of amino acids, carbon metabolism, ABC transporter, phosphotransferase system (PTS), ribosome, starch and sucrose metabolism, quorum sensing, glycolysis/gluconeogenesis, and purine metabolism.

### 3.3. Analysis of DEPs

**Figure 3:**
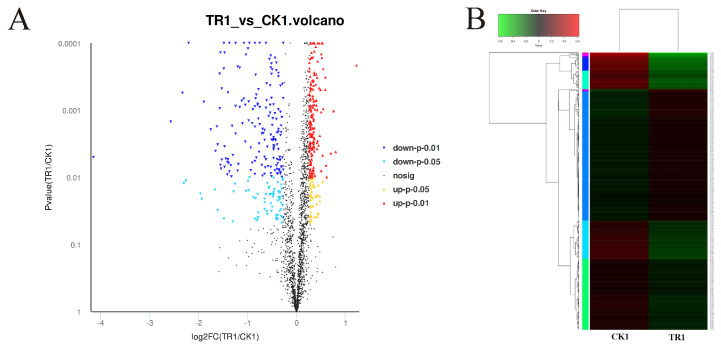
DEPs of LM cells analyzed between the treated group (treated with linalool) and the control group (untreated with linalool). (**A**) Volcano plot of DEPs. The abscissa represents the fold change value of the difference between control and treated samples. The difference value is obtained by dividing the expression level of control sample by treated sample, and this value is logarithmized. The ordinate represents *p*-value (by the analysis of statistical *t*-test) of the difference of protein expressions. The smaller the *p*-value, the more significant the difference in protein expression. Each point represents a specific protein: the yellow point (significantly up-regulated, *p* < 0.05), the red point (significantly up-regulated, *p* < 0.01), the light blue point (significantly down-regulated, *p* < 0.05), the blue point (significantly down-regulated, *p* < 0.01), and the black dots (non-significantly different proteins, *p* > 0.05). (**B**) Heat map of DEPs. The left (CK1, control group) and right columns (TR1, treated group) represent control and treated groups, respectively. Each row represents a protein. Red and green colors represent the high and low expression levels of the protein, respectively. On the left is the dendrogram of protein clustering, and on the right is the name of the protein.

Through the differential expression analysis of all the identified proteins between the linalool treatment group and control group, a total of 445 DEPs were screened out, including 211 up-regulated and 234 down-regulated proteins. As shown in Figure 3, the data of the DEPs were converted to volcano plots and heat maps for more intuitionistic comparative analysis.

### 3.4. The GO Enrichment Analysis

The enrichment analysis method is usually used to analyze whether a group of proteins has appeared on a certain functional node in a certain pathway; the aim is to make the annotation analysis from a single protein to a protein set. Enrichment analysis improves the reliability of research and can screen out the biological processes most relevant to biological phenomena. Therefore, GO enrichment analysis was performed to analyze the functional enrichment of differential proteins and clarify the differences between treated and control groups at the functional level.

**Figure 4:**
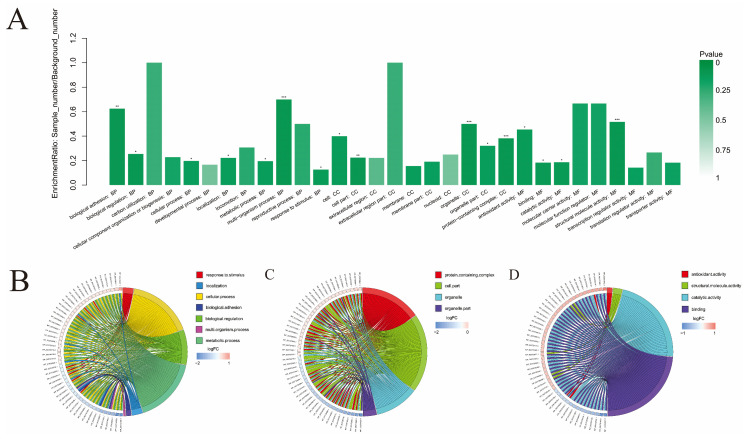
The GO enrichment analyzed between the treated group (treated with linalool) and the control group (untreated with linalool). (**A**) Histogram of GO enrichment of DEPs. Each column represents a GO term, and the abscissa represents the name and category of GO. The ordinate represents the enrichment rate. The color represents the significance of enrichment. *p* < 0.05, *p* < 0.01, *p* < 0.001 are marked as *, **, and ***, respectively. Subfigures (**B**–**D**) are chord diagrams of the GO enrichment of DEPs, respectively showing the (**B**) different proteins participating in specific functions in the three GO categories of biological process, (**C**) cell composition, (**D**) and molecular function.

As shown in Figure 4, a total of 31 significantly enriched functional categories were obtained. Among them, there were 12 categories in “Biological Process”, including biological adhesion (GO:0022610), biological regulation (GO:0065007), carbon utilization (GO:0015976), cellular component organization or biogenesis, (GO:0071840), cellular process (GO:0009987), developmental process (GO:0032502), localization (GO:0051179), locomotion (GO:0040011), metabolic process (GO:0008152), multi-organism process (GO:0051704), reproductive process (GO:0022414), and response to stimulus (GO: 0050896). There were 10 categories in “Cell Component”, including cell (GO:0005623), cell part (GO:0044464), extracellular region (GO:0005576), extracellular region part (GO:0044421), membrane (GO:0016020), membrane part (GO:0044425), nucleoid (GO:0009295), organelle (GO:0043226), organelle part (GO:0044422), and protein-containing complex (GO:0032991). There were nine categories in “Molecular Function”, including antioxidant activity (GO:0016209), binding (GO:0005488), catalytic activity (GO:0003824), molecular carrier activity (GO:0140104), molecular function regulator (GO:0098772), structural molecule activity (GO:0005198), transcription regulator activity (GO:0140110), translation regulator activity (GO:0045182), and transporter activity (GO:0005215).

### 3.5. The KEGG Enrichment Analysis

**Figure 5:**
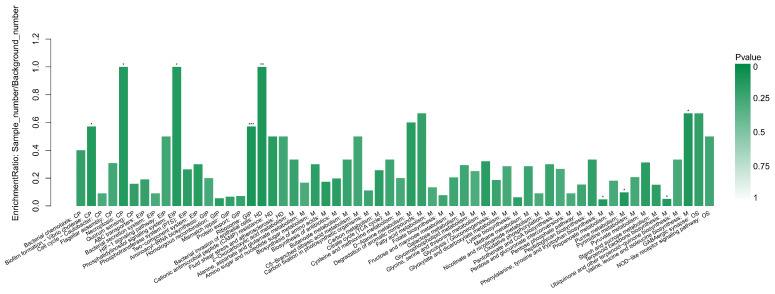
The enrichment of the DEPs in KEGG pathways analyzed between the treated group (treated with linalool) and the control group (untreated with linalool). Each column represents a pathway, and the abscissa represents the name and classification of the pathway: CP (cellular process), EIP (environmental information processing), GIP (genetic information processing), HD (human diseases), M (metabolism), and OS (organismal systems). The height of the column or the ordinate represents the enrichment rate. The color represents the significance of enrichment. *p* < 0.05, *p* < 0.01, and *p* < 0.001 are marked as *, **, and ***, respectively.

In addition to the GO enrichment analysis, KEGG pathway enrichment analysis was also performed to analyze the biological pathways involved in DEPs in this study. As shown in Figure 5, through KEGG enrichment analysis, a total of 60 significantly enriched biological pathways were identified. Among them, there were 6 pathways in the “Cell Process” category, 6 pathways in the “Environmental Information Processing” category, 3 pathways in the “Human Disease” category, 40 pathways in the “Metabolism” category, and 2 pathways in the “Organic System” category.

### 3.6. Flow Cytometry Analysis

**Figure 6:**
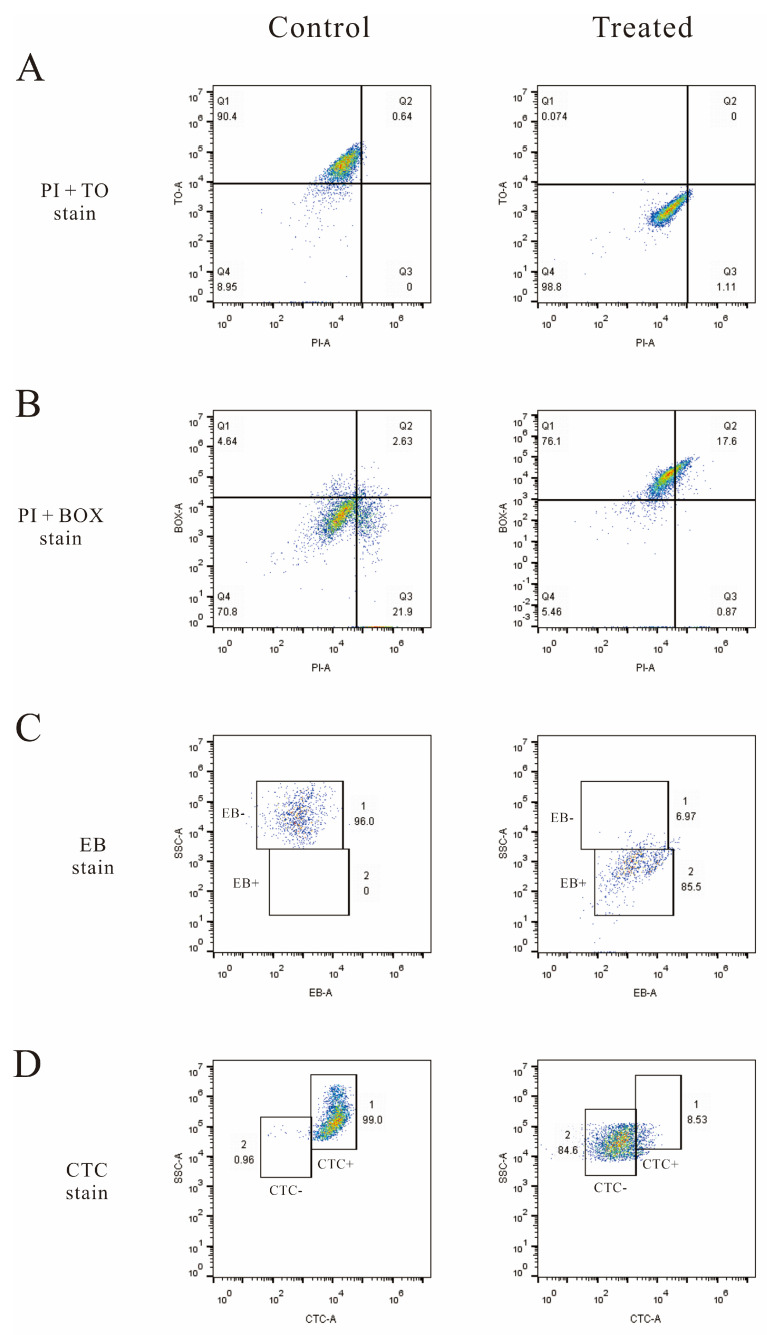
Fluorescence density plots of LM treated (treated) and untreated (control) with linalool stained with (**A**) PI and TO, (**B**) PI and BOX, (**C**) EB, and (**D**) CTC. For subfigures (**A**) and (**B**), the vertical and horizontal axis indicate the fluorescence intensity. The percentages of the cell population in each gate are demonstrated in the four corners of each plot.

Five fluorescent dyes (TO, PI, BOX, EB, and CTC) were used to evaluate several vital biological functions in LM cells by flow cytometry analysis. Membrane integrity was evaluated by double staining of TO and PI as shown in Figure 6A. In the control group, 90.4% of the cells were located in plot Q1 (TO+ and PI−), which represented cells with an intact cell membrane. By contrast, in the treated groups, 98.8% of the cells were located in plot Q4 (TO− and PI−), which represented cells with damaged DNA or RNA.

Membrane potential was evaluated by double staining of BOX and PI as shown in Figure 6B. In the control group, 70.8% of the cells were located in plot Q4 (BOX− and PI−), which represented cells with a polarized membrane. By contrast, in the treated groups, 76.1% and 17.6% of the cells were located in plot Q1 (BOX+ and PI−) and plot Q1 (BOX+ and PI+), which represented cells with depolarized nonpermeabilized and permeabilized membranes, respectively. In total, after the treatment of linalool, 93.7% cells became depolarized.

The efflux activity was evaluated by EB staining as shown in Figure 6C. EB− represents the efflux pump functioning properly, while EB+ represents the malfunction of the efflux pump. The percentages of EB+ cells in the control and treated groups were 2% and 85.5%, respectively, and the percentages of EB− cells in these two groups were 96% and 6.97% respectively. These data show that the efflux pump of 85.5% of the cells became malfunctioning after the treatment of linalool.

Respiratory activity was evaluated by CTC staining as shown in Figure 6D. CTC+ and CTC− represent respiratory active and inactive cells, respectively. The percentage of CTC+ cells in the control and treated groups were 99% and 8.53%, and the percentage of CTC− cells in these two groups were 0.96% and 84.6%.

## 4. Discussion

### 4.1. Proteomic Technology Used in the Antimicrobial Research Field

In this study, iTRAQ-based quantitative proteomics analysis was performed to identify proteins differentially expressed between the linalool treated group and the untreated group. Finally, a total of 445 DEPs were screened out, including 211 up-regulated and 234 down-regulated proteins which participated in different biological functions and pathways.

Except for our study, there have been few other studies focused on the theme of “changes of microbial proteomics after essential oils treatment”; some of these studies are summarized as follows. Xu et al. [32] studied the proteomic changes of *Botrytis cinerea* after tea tree oil treatment, finding a total of 718 DEPs, of which 17 were up-regulated and 701 were down-regulated. These proteins were annotated to 30 GO categories and 133 KEGG pathways, including glycolysis, tricarboxylic acid cycle, and purine metabolism pathways. Hu et al. [33] screened out a total of 745 DEPs of LM after thyme essential oil treatment, of which 246, 45, and 309 proteins were involved in biological processes, cellular components, and molecular functions, respectively. Meanwhile, these proteins participated in 86 KEGG pathways, such as flagella assembly and chemotaxis. Yang et al. [13] demonstrated the proteomic changes of *Klebsiella pneumoniae* after treatment by lavender essential oil. A total of 135 DEPs were found, of which 57 were up-regulated and 78 were down-regulated; they were annotated to 30 GO categories and 133 KEGG pathways. Moreover, 35.78%, 34.01%, 8.44%, and 8.84% of these proteins were involved in cellular processes, metabolic processes, cellular component structure, and stress response, respectively; 58.27%, 17.53%, 13.33%, and 6.17% were involved in the cytoplasm, cell membrane, protein complex, and ribosomal proteins, respectively; and 44.40% and 42.28% were involved in binding and catalytic activity.

Although there are many differences between the above studies and ours, there are also some similarities, such as changes in glycolysis and other metabolic pathways. These differences might be caused by different proteomic technologies, types of essential oils, and the different microbial strains used. Proteomic technology has been proved to be an efficient, fast, and useful approach to identify differentially expressed proteins in various types of microorganisms.

### 4.2. Further Analysis of Important Functions in GO Enrichment Analysis

Several categories relevant to “cell component” were enriched as shown in Figure 4A. Firstly, the enrichment of “cell (GO:0005623)” and “cell part (GO:0044464)” indicated that the structure and component of bacterial cells were altered significantly after linalool treatment. Secondly, the enrichment of “membrane (GO:0016020)” and “membrane part (GO:0044425)” was in accordance with the results of SEM and TEM in our previous study, which showed obvious damage of the cell membrane [15]. Thirdly, the enrichment of “nucleoid (GO:0009295), organelle (GO:0043226), and organelle part (GO:0044422)” echoed the data of the flow cytometry analysis, which proved that the DNA or RNA (main part of the nucleoid) might be destroyed by linalool treatment; meanwhile, “organelle part” also echoed the enrichment of the “ribosome pathway (ko03010)” in KEGG analysis. To sum up, all the above data implied that the cell membrane, nucleoid, and ribosome may be the potential targets of linalool.

As shown in Figure 4A, some categories related to “molecular function” were also enriched. Initially, the enrichment of “structure molecule activity (GO:0005198)” meant that the action of molecules which contributes to the structural integrity or assembly of a complex significantly changed. Furthermore, the enrichment of both “transcription regulator activity (GO:0140110)” and “translation regulator activity (GO:0045182)” indicated the gene expression and polypeptide synthesis were significantly influenced by linalool, which was further evidence showing that the nucleoid and ribosome might be the potential targets.

Some categories relevant to “biological process” were also enriched as shown in Figure 4A. Primarily, the enrichment of “response to stimulus (GO:0050896)” indicated that cells may produce many adaptive responses to meet the challenge of the stimulus (linalool). Further, the enrichment of “biological adhesion (GO:0022610)” might be related to two proteins (InlA and InlB) as shown in the “bacterial invasion of epithelial cells (ko05100)” pathway in KEGG analysis. These two proteins are surface invasions, which could promote the uptake of LM by host cells [34] and enhance the adherence of LM to a glass surface [35]. Moreover, the enrichment of “reproductive process (GO:0022414)” suggested that linalool treatment might influence the reproduction of cells, which was also shown in our previous study [15].

### 4.3. Further Analysis of Important Pathways in KEGG Enrichment Analysis

First of all, as shown in the “peptidoglycan biosynthesis (ko00550)” pathway in Figure S1, several proteins related to peptidoglycan biosynthesis were down-regulated, which indicated that the biosynthesis of peptidoglycan was reduced by linalool treatment. Two kinds of penicillin-binding proteins (PBPs), PBP 2 and PBP 1a, were down-regulated. PBPs are a major class of enzymes related to peptidoglycan synthesis, which were identified as targets of β-lactam antibiotics (such as penicillin); they inactivate the crosslinking domains of peptidoglycan synthesis covalently [36]. Peptidoglycan is a vital component of cell walls, maintaining the morphology and viability of bacterial cells [37]. It has been proven that peptidoglycan is the target of many antimicrobial drugs, such as penicillin [36] and cephalosporin [38]. Thus, our results imply that peptidoglycan and cell walls might be another important drug target of linalool.

Next, two peptidoglycan enzymes, AmiA and AmiC, were down-regulated as shown in Figure S2. AmiA and AmiC are N-acetylmuramyl-l-alanine amidases that move side chains away from bacterial peptidoglycan through cleaving the amide bond between peptides and N-acetylmuramic acids [39], which are crucial for septal splitting and the separation of daughter cells [40]. Thus, the down-regulation of these two proteins indicated that the cell division was inhibited by linalool treatment, and this phenomenon might also prove the changing of the “reproductive process” in the GO enrichment analysis.

Moreover, many proteins relevant to the structure and function of ribosome were significantly up- or down-regulated as shown in the “ribosome (ko03010)” pathway in Figure S3, which indicates that the function of ribosome was obviously affected by linalool treatment. These were consistent with our results in GO enrichment analysis, which again suggested ribosomes were the target of linalool.

To sum up, based on our results from the GO and KEGG enrichment analysis, cell membranes, cell walls, nucleoids, and ribosomes might be the main targets of linalool against LM. These results also proved the multi-target effects of linalool, which is one of its most important advantages compared to traditional antibiotics.

## 5. Conclusions

In this study, iTRAQ-based quantitative proteomics sequencing was carried out to unravel the mode of action of linalool against LM at the protein level. A total of 445 DEPs (including 211 up-regulated and 234 down-regulated proteins) were screened out between the linalool treatment group and the control group. GO and KEGG enrichment analysis implied that cell membranes, cell walls, nucleoids, and ribosomes might be the targets of linalool against LM, which was also supported by the results of the flow cytometry analysis. In the future, we will focus on each of these targets and investigate the specific mechanisms. Meanwhile, the application of linalool for the prevention of LM in foods or food facilities should also be explored.

## Supplementary Materials

The following are available online at www.mdpi.com/article/10.3390/foods10102449/s1, Figure S1: Peptidoglycan biosynthesis pathway from the KEGG analysis. Figure S2: AmiA and AmiC annotation from the KEGG analysis. Figure S3: Ribosome pathway from the KEGG annotation analysis.
