# Supplementary material for: Global Proteomic Analysis of Listeria monocytogenes’ Response to Linalool
Source: Foods. 2021 Oct 14;10(10):2449. doi: 10.3390/foods10102449 (PMC8535586; doi:10.3390/foods10102449)
Supplement: Supplementary file 1 [file foods-10-02449-s001.zip › foods-1380441-supplementary.pdf]

# Figure S1

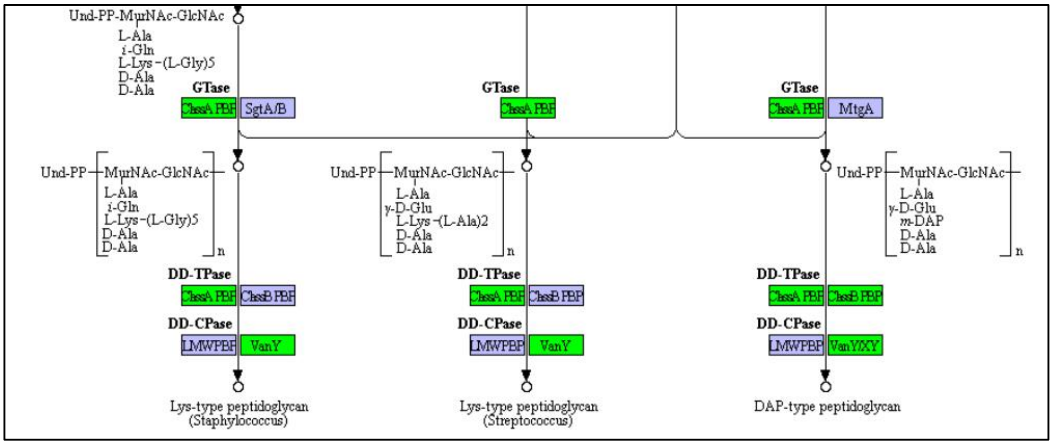


**Figure S1**. Peptidoglycan biosynthesis pathway from the KEGG analysis. The genes with red/green borders belong to the differential genes detected by RNA-sequencing, in which red represents the up-regulated genes and green represents the down-regulated genes.

# Figure S2

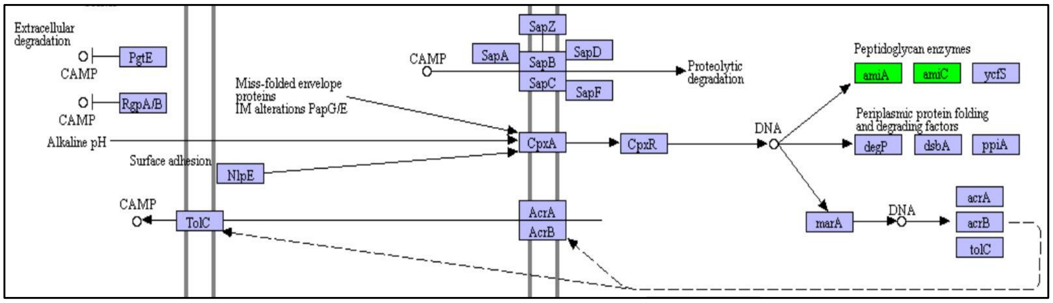


**Figure S2**. AmiA and AmiC annotation from the KEGG analysis. The genes with red/green borders belong to the differential genes detected by RNA-sequencing, in which red represents the up-regulated genes and green represents the down-regulated genes.

# Figure S3

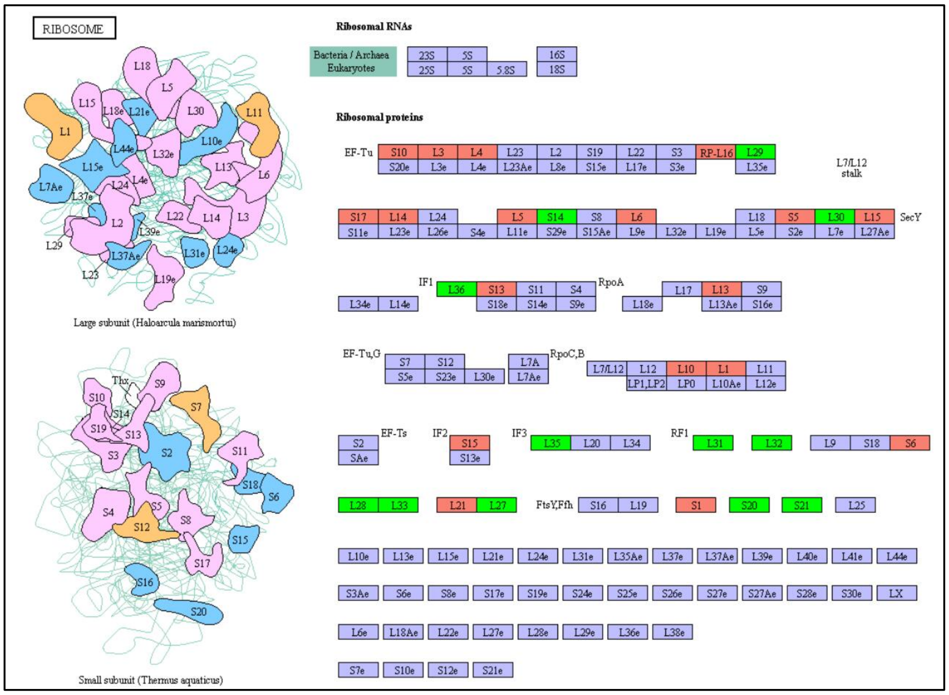

**Figure S3**. Ribosome pathway from the KEGG annotation analysis. The genes with red/green borders belong to the differential genes detected by RNA-sequencing, in which red represents the up-regulated genes and green represents the down-regulated genes.
